# Resident obstetricians’ awareness of the oral health component in management of nausea and vomiting in pregnancy

**DOI:** 10.1186/s12884-014-0388-9

**Published:** 2014-11-25

**Authors:** Joan Enabulele, Louis Ibhawoh

**Affiliations:** Department of Restorative Dentistry, University of Benin, Benin City, Edo State Nigeria

**Keywords:** Nausea, Vomiting, Oral health, Pregnancy

## Abstract

**Background:**

Nausea and vomiting are common in early pregnancy in 50-90% of pregnant women and resolves in all but 10% of these women. Many obstetricians encounter this problem and should be familiar with the probable outcomes, current treatment options and oral health component of its management. This study assessed the awareness of obstetrics residents of the oral health component of management of nausea and vomiting in pregnancy.

**Methods:**

This study was carried out among resident doctors in Obstetrics and Gynaecology in Nigeria. A pre-tested, self-administered questionnaire was used for the data collection. The data collected were analyzed using the Statistical Package for Social Science (SPSS) version 17.0. Non parametric analysis in the form of chi square was carried out to test for statistical significance with P value <0.05 considered statistically significant.

**Results:**

A total of 200 questionnaires were administered while 186 were filled and returned, giving a response rate of 93%, comprising 21.5% senior residents and 78.5% junior residents. Most of the respondents agreed that oral health is important in pregnancy. A majority (58%) also thought that oral health complaints in pregnancy were not normal. Fifty-seven percent of the respondents neither assessed teeth and gums of pregnant women for problems during ante-natal care nor educated them on care that would improve their oral health. Majority (95.7%) of the respondents assisted pregnant women with dealing with nausea and vomiting but were not aware of the oral health component of its management.

**Conclusion:**

Oral health component in the management of nausea and vomiting in pregnancy has been largely neglected in obstetric care. It is pertinent that ante-natal health care providers receive adequate education on perinatal oral health care.

## Background

Nausea and vomiting are common in early pregnancy (4^th^ to 7^th^ week after last menstrual cycle) in 50-90% of pregnant women [1,2] and resolves in all but 10% of these women [1]. Many obstetricians encounter this problem and should be familiar with the probable outcomes and current treatment options [2]. Nausea and vomiting of pregnancy are usually self- limiting conditions [3]. Women with uncomplicated nausea and vomiting of pregnancy have been noted to have improved pregnancy outcomes compared to women with complicated nausea and vomiting of pregnancy [4,5].

Improving the oral health of pregnant women prevents complications of dental diseases during pregnancy [6]. Maternal oral diseases have been associated with pre-term births [7-10], development of pre-eclampsia [11,12] and delivery of a “small-for-gestational-age” infant [7,9,10]. This could lead to infant mortality and morbidity. Nausea and vomiting may cause a woman to avoid routine oral health practices such as tooth brushing and flossing. This could lead to dental caries [13], gingivitis [14] which is reported to be the most common oral disease during pregnancy with a prevalence rate of 60-75% [15] and periodontitis [14], a bacterial infection which is detectable in up to 30% of pregnant women which has been linked with adverse pregnancy outcomes such as pre-term delivery and low birth weight outcome among infants [16-21]. Food cravings induced by pregnancy may lead to frequent consumption of foods high in carbohydrates and therefore increase the risk of dental caries [22]. Tooth erosion can result from nausea and vomiting of pregnancy [23] because of the hydrochloric acid content of regurgitated gastric juice [24]. In addition, the buffering capacity of saliva could change during pregnancy thus creating a more acidic environment [25]. This coupled with frequent vomiting increases the tendency for tooth erosion. The effects of tooth erosion such as marked tooth sensitivity may last beyond the duration of the pregnancy.

The management of nausea and vomiting is dependent upon its impact on the affected woman’s health, quality of life and the safety of maternal treatment on the developing fetus. Management includes both non-pharmacological and pharmacological therapy. Professional guidelines and policy statements have been formulated for perinatal oral health care [6,26-31]. To help reduce the erosion of tooth surfaces in women experiencing frequent nausea and vomiting, the following guidelines have been proposed: eating small quantities of nutritious yet non-cariogenic foods and/or snacks which are rich in protein such as cheese throughout the day, using a solution of a teaspoon of baking soda (sodium bicarbonate) in a cup of water for mouth rinses, avoiding tooth brushing immediately after vomiting as the effect of erosion can be exacerbated by brushing an already demineralized tooth surface. Other guidelines include using gentle tooth brushing with medium-texture bristle toothbrush and fluoride toothpaste twice daily when nausea is minimal to prevent damage to demineralized tooth surfaces and using a fluoride-containing mouth rinse immediately before bedtime to help remineralize teeth.

There is limited access to dentists worldwide, especially in low income economies making a high proportion of women unable to see a dentist during pregnancy. Therefore, oral health promotion must be incorporated into prenatal medical care to help bridge this gap. It is important that while managing pregnant women with nausea and vomiting, the oral health component is taken into consideration by the obstetricians. This study was designed to assess the awareness of resident obstetricians to the oral health component of management of nausea and vomiting in pregnancy.

## Methods

This study was carried out among resident doctors in Obstetrics and Gynaecology in Nigeria preparing for the various levels of the fellowship examinations of the West Africa College of Surgeons and the National Postgraduate Medical College of Nigeria, who attended the revision course in Calabar, Cross River State in September, 2011. The study was exempted from ethical approval by the College of Medical Sciences Research Ethics Committee, University of Benin, Benin City, Edo State Nigeria (CMS/PO/109/Vol.2/030) however, the study was carried out in compliance with regulations governing the protection of human subjects in medical research and written informed consent was obtained from the participants prior to commencement of the study.

Prior to the actual study, pre-testing of the questionnaire was completed on 20 clinical students undergoing Obstetric clinical rotations. This was to enable the researchers determine the appropriateness of the questionnaire as a tool for collecting the required information. The questionnaire consisted of 3 sections which sought information on respondents’ demography, attitude and practice regarding oral health in pregnancy and management of nausea and vomiting in pregnancy.

The data collected were analyzed using the Statistical Package for Social Science (SPSS) version 17.0. The results were presented as bar chart and cross tabulations. Non parametric analysis in the form of chi square was carried out to test for statistical significance with P value <0.05 considered statistically significant.

## Results

A total of 200 questionnaires were administered while 186 were filled and returned, giving a response rate of 93%, comprising 21.5% senior residents and 78.5% residents with an average of 6.46 ± 3.7 years post-graduation experience. The male: female ratio was 5.6:1. Almost half (49.5%) of the respondents were between the ages of 31-35 years (Table 1).

**Table 1:** **Demographic characteristics of the respondents**

	**Gender**		
	**Male n (%)**	**Female n (%)**	**Total n (%)**
Age (years)			
26-30	16 (10.1)	2 (7.1)	18 (9.7)
31-35	80 (50.6)	12 (42.9)	92 (49.5)
36-40	40 (25.3)	12 42.9)	52 (28.0)
41-45	16 (10.1)	0 (0.0)	16 (8.6)
46-50	4 (2.5)	2 (7.1)	6 (3.2)
>50	2 (1.3)	0 (0.0)	2 (1.1)
Total	158 (100.0)	28 (100.0)	186 (100.0)

**Figure 1:**
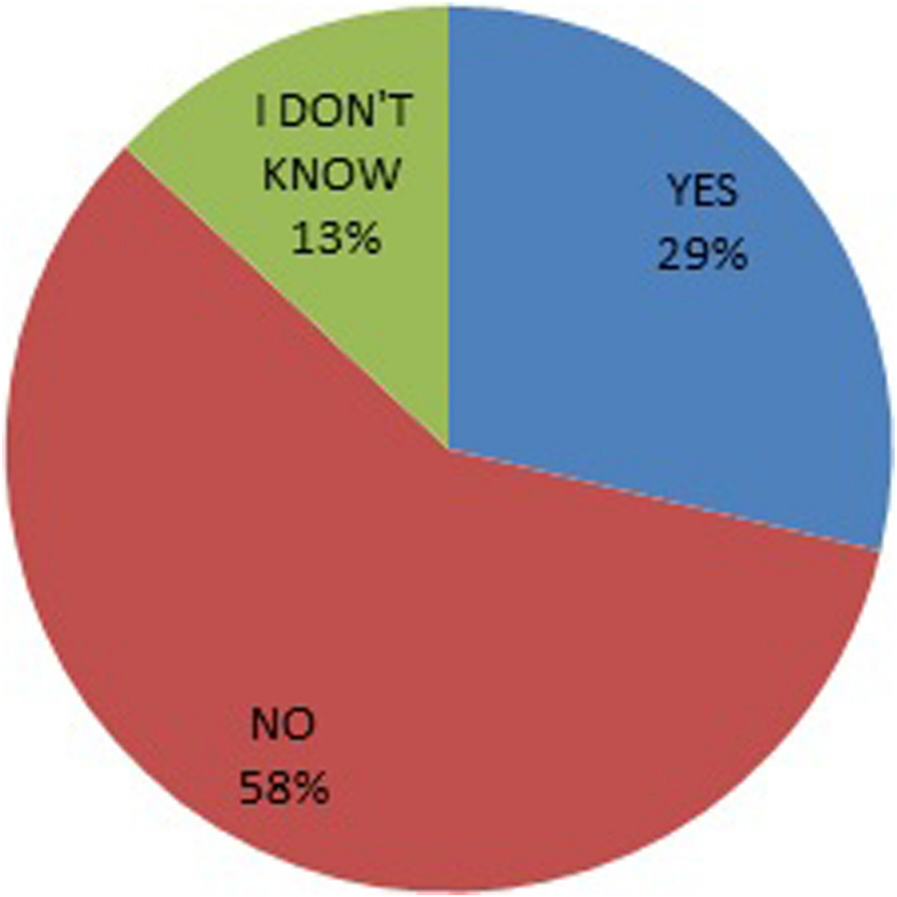
**Perception of respondents whether oral health complaints in pregnancy are normal.**

Most (97.9%) of the respondents agreed that oral health is important in pregnancy with 53.8% strongly agreeing. A majority (58%) also thought that oral health complaints in pregnancy were disorders associated with pregnancy (Figure 1). Fifty-seven percent of the respondents neither assessed teeth and gums of pregnant women for problems during ante-natal care nor educated them on care that would improve their oral health.

Majority (95.7%) of the respondents assisted pregnant women with dealing with nausea and vomiting by giving advice regarding non-pharmacological means of dealing with nausea and vomiting in pregnancy but were not aware of the oral health component of its management.

More than half (53.8%) of the respondents advised pregnant women to eat small amounts of nutritious but non-cariogenic foods throughout the day. Only 7.5% advised pregnant women on the use of a solution of a teaspoon of baking soda (sodium bicarbonate) in a cup of water for mouth rinses while, 46.2% advised them to rinse with clean water only after vomiting to neutralize the acid from the vomitus. Tooth brushing immediately after vomiting was incorrectly advised by 19.1% of the respondents with 22.9% of junior residents and 5.3% of senior residents advising pregnant women incorrectly to brush after vomiting though this was not statistically significant (Table 2). Less than half (46.2%) of the respondents advised the pregnant women on gentle tooth brushing with medium-texture bristle toothbrush and fluoride toothpaste twice daily when nausea was minimal to prevent damage to demineralised tooth surfaces. There were no significantly different responses between male and female responses and between physicians from different age groups.

**Table 2:** **Status of respondents vs giving advice on tooth brushing immediately after an episode of vomiting**

	**Advice on tooth brushing immediately after vomiting**		
	**Yes n (%)**	*No n (%)	**Total n (%)**
Status			
Resident	32 (22.9)	108 (77.1)	140 (100.0)
Senior resident	2 (5.3)	36 (94.7)	38 (100.0)
Total	34 (19.1)	144 (80.9)	178 (100.0)

P = 0.08.

*“No” brushing is the correct response.

**Figure 2:**
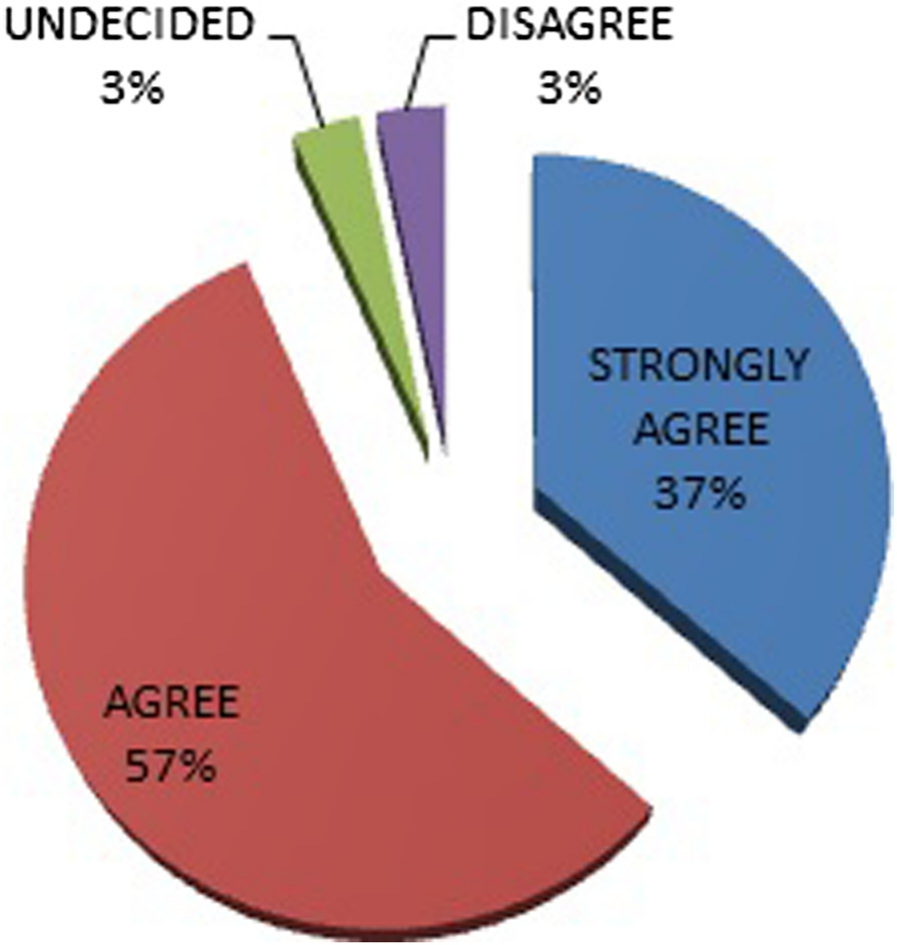
**Need for obstetricians to be educated on oral health care.**

A vast majority 93.5% thought obstetricians should be educated on oral health care in pregnancy with 36.6% strongly agreeing (Figure 2).

## Discussion

Nausea and vomiting during pregnancy can cause extensive erosion of the tooth surface leading to deteriorating oral health status [16] which has been associated with adverse pregnancy outcomes such as pre-term births [7-10], development of pre-eclampsia [11,12] and delivery of a “small-for-gestational-age” infant [7,9,10]. It is imperative therefore; that the management of nausea and vomiting should involve measures targeted at reducing the effects of the hydrochloric acid content of gastric juice on the teeth as well as improve oral hygiene practices to prevent poor oral health. This study confirms the importance attributed to oral health in pregnancy among obstetricians as was reported in a previous study [32]. However, as high as 42% of them still thought that oral health complaints were not disorders associated with pregnancy or unsure if they were disorders associated with pregnancy. This goes to buttress the fact that though it has been reported that a substantial proportion of pregnant women report experiencing oral health problems during pregnancy, most of such are not perceived by antenatal health care providers as disorders in pregnancy [33] that warrant evaluation and possible intervention. A gap has been recognized between the need for provision of good oral health and the necessary action to provide the best possible total care for pregnant women [34].

A large proportion of obstetricians in this study neither assessed teeth and gums of pregnant women for problems during ante-natal care nor educated them on care that would improve their oral health including referrals to dentist for routine checks. Many pre-natal providers fail to refer their patients regularly for dental consultation [35] especially as oral health screening is not routinely done in many ante-natal clinics and there are no standard policies to ensure that all pregnant women are routinely screened and referred for dental treatment [35,36]. There is a need for obstetric organizations that make policies to get up-to-date advice from dental professionals to publish guidelines that are consistent with best practice. Once there are guidelines, it is important that resident obstetricians are trained on them.

Assisting pregnant women with dealing with nausea and vomiting is very common given the fact that nausea and vomiting are common in early pregnancy in 50 to 90% of pregnant women [1,2]. This was corroborated in this study with majority of the respondents reporting that they assisted pregnant women. They gave advice regarding non-pharmacological means of dealing with nausea and vomiting in pregnancy although, they were not conscious of the oral health component of management of nausea and vomiting in pregnancy.

Official obstetric guidelines need to adequately emphasize the oral health issues related to nausea and vomiting in pregnancy and ways to reduce the effect. This study suggests that the use of a solution of a teaspoon of baking soda (sodium bicarbonate) in a cup of water for mouth rinses, one of the non-pharmacological methods of dealing with nausea and vomiting is not popular. The need to neutralize the acid from the vomitus is highlighted with respondents advising pregnant women to rinse with clean water only after vomiting. Tooth brushing immediately after vomiting is not recommended, however resident obstetricians were giving pregnant women incorrect advice.

Oral health assessment is not routinely incorporated into ante-natal visits [26,37] so pregnant women don’t get to know that gentle tooth brushing using medium-texture bristle toothbrush and fluoride toothpaste twice daily to prevent damage to demineralized tooth surfaces.

The dearth of oral health knowledge among ante natal care providers has been highlighted and the need for formal and informal training identified [38]. Obstetricians in this study acknowledged the need for obstetricians to be educated on oral health care in pregnancy with probable improvement in oral health assessment and management (including referrals to dentists when necessary) during pregnancy.

The authors acknowledge a double barreled question (Do you advise pregnant women to eat small amounts of nutritious but non-cariogenic foods throughout the day?) and don’t have the data of what proportion advised pregnant women to eat small amounts of nutritious foods which are commonly cariogenic such as biscuits throughout the day separately from the proportion of respondents that advised pregnant women to eat non-cariogenic foods throughout the day.

## Conclusion

The oral health component in the management of nausea and vomiting in pregnancy has been largely neglected in obstetric care. A substantial proportion of obstetric residents are giving pregnant women incorrect advice on dietary and oral hygiene practices that could be harmful to their oral health and pregnancy. It is imperative therefore, that in the overriding interest of better total care for pregnant women, ante-natal health care givers receive adequate education on perinatal oral health.

## References

[1] Gadsby R, Barnie-Adsheed AM, Jagger C (1993). A prospective study of nausea and vomiting during pregnancy. Br J Gen Pract.

[2] Jarvis S, Nelson-Piercy C (2011). Management of nausea and vomiting in pregnancy. Br Med J.

[3] Quinlan JD, Hill DA (2003). Nausea and vomiting of pregnancy. Am Fam Phy.

[4] Brandes JM (1967). First trimester nausea and vomiting as related to outcome of pregnancy. Obstet Gynecol.

[5] Jarnfelt-Samsioe A, Samsioe G, Velinder GM (1983). Nausea and vomiting in pregnancy- a contribution to its epidemiology. Gynecol Obstet Invest.

[6] Family Health Bureau, Ministry of Health (2009). Oral Health Care during Pregnancy: Practice Guidelines.

[7] Offenbacher S, Katz V, Fertila G, Collins J, Boyd D, Maynor G, McKaig R, Beck J (1996). Periodontal infection as a possible risk factor for pre-term low birth weight. J Periodontol.

[8] Jeff Coat MK, Geus NC, Reddy MS, Cliver SP, Goldeneg RL, Hauth JC (2001). Periodontal infection and preterm birth: Results of a prospective study. J Am Dent Assoc.

[9] Umoh AO, Ojehanon PI, Savage KO (2013). Effect of maternal periodontal status on birth weight. Eur J Gen Dent.

[10] Umoh AO, Ojehanon PI, Akhionbare O, Savage KO (2012). Relationship between maternal oral hygiene status with low birth weight and preterm deliveries. Nig Dent J.

[11] Boggess KA, Lieff S, Murtha AP, Moss K, Beck J, Offenbacher S (2003). Maternal periodontal disease is associated with an increased risk for pre-eclampsia. Obstet Gynecol.

[12] Canakci V, Canakci CF, Canakci H, Canakci E, Cicek T, Ingec M, Ozgoz M, Demir T, Dilsiz A, Yagiz H (2004). Periodontal disease as a risk factor for pre-eclampsia: a case control study. Aust NZJ Obstet Gynaecol.

[13] **Improving access to perinatal oral health care: Strategies and considerations for health plans.** [https://www.cdhp.org/resources/237-improving-access-to-perinatal-oral-health-care-strategies-considerations-for-health-plans]

[14] Kandan PM, Menaga V, Kumar RRR (2011). Oral health in pregnancy (Guidelines to gynaecologists, general physicians and oral health care providers). J Pakistan Med Assoc.

[15] Silk H, Douglass AB, Douglass JM, Silk L (2008). Oral health during pregnancy. Am Fam Physician.

[16] **Oral health care for pregnant women.** South Carolina oral health advisory council coalition 2008. [http://webcache.googleusercontent.com/search?q=cache:cSob0ohxEYUJ:www.scdhec.gov/library/CR-009437.pdf+&cd=1&hl=en&ct=clnk&gl=ng&client=firefox-a]

[17] Bobetsis YA, Barros SP, Offenbacher S (2006). Exploring the relationship between periodontal disease and pregnancy complications. J Am Dent Assoc.

[18] Sacco G, Carmagnola D, Abati S, Luglio PF, Ottonlenghi L, Villa A, Maida C, Campus G (2008). Periodontal disease and preterm relationship: a review of the literature. Minerva Stomatol.

[19] Clothier B, Stringer M, Jeff Coat MK (2007). Periodontal disease and pregnancy outcomes: exposure, risks and interventions. Best Pract Res Clin Obstet Gynaecol.

[20] Yeo BK, Lim LP, Paquette DW, Williams RC (2005). Periodontal disease- the emergence of a risk for systemic conditions: pre-term low birth weight. Ann Acad Med Singapore.

[21] Champagne CM, Madianos P, Lieff S, Murtha AP, Beck JD, Offenbacher S (2000). Periodontal medicine: emerging concepts in pregnancy outcome. J Int Acad Periodontol.

[22] Ziegler J, Mobley CC: **Pregnancy, child nutrition and oral health.** [http://www.google.com.ng/url?sa=t&rct=j&q=&esrc=s&source=web&cd=3&cad=rja&uact=8&ved=0CCoQFjAC&url=http%3A%2F%2Fwww.springer.com%2Fcda%2Fcontent%2Fdocument%2Fcda_downloaddocument%2F9781607614890-c2.pdf%3FSGWID%3D0-0-45-1451207-p176744429&ei=7kd2VJK8Hsa9ygOnh4GYDQ&usg=AFQjCNHxKQK4COJbUB5-yZ_m7yGTME11iw&sig2=RUzi15Z-mD6cZ72fGvfVJA&bvm=bv.80642063,d.bGQ]

[23] Kumar J, Samelson R. Eds. **Oral Health Care During Pregnancy and Early Childhood: Practice Guidelines.** [https://www.health.ny.gov/publications/0824.pdf]

[24] Johansson A, Omar R, Carlsson GE, Johansson A (2012). Dental erosion and its growing importance in clinical practice: from past to present. Int J Dent.

[25] Laine MA (2002). Effect of pregnancy on periodontal and dental health. Acta Odontol Scand.

[26] Kumar J, Samelson R (2009). Oral health care during pregnancy recommendations for oral health professionals. New York State Dent J.

[27] Hujoel PP, Bollen A, Noonan CJ, Del Aguila MA (2004). Antepartum dental radiography and infant low birth weight. J Am Med Assoc.

[28] American Academy of Pediatrics and American Congress of Obstetricians and Gynaecologists (2007). Guidelines for Perinatal Care.

[29] American Academy of Periodontology: **Statement regarding periodontal management of the pregnant patient, 2004.** [http://www.albertadentalspecialists.ca/resources/32-AAPPeriodontalManagementofaPregnantPatient.pdf] 10.1902/jop.2004.75.3.495 15088891

[30] American Academy of Pediatrics: **Policy Statement on oral health risk assessment, timing and establishment of the dental home.** [http://pediatrics.aappublications.org/content/111/5/1113] 10.1542/peds.111.5.1113 12728101

[31] American Academy of Pediatric Dentistry: **Guideline on perinatal oral health care.** [http://www.aapd.org/media/Policies_Guidelines/G_PerinatalOralHealthCare.pdf]

[32] Pahl S, Thakur R, Madku K, Paul ST, Gadichela P (2013). Oral health coalition: knowledge, attitude, practice and behavior among gynaecologists and dental practitioners. J Int Oral Health.

[33] Mwangosi IEAT, Kiango MM (2012). Oral health experience during pregnancy and dental service utilization in Bariadi district, Tanzania. Tanz J Health Res.

[34] Enabulele JE, Ibhawoh LO (2012). Perceptions and practices regarding perinatal oral health: a survey of ante-natal care physicians. Nig Res J Clin Sci.

[35] Mills LW, Moses DT (2002). Oral health during pregnancy. MCN Am J Matern Child Nurs.

[36] Zenata RL, Fernandes KB, Navamo PS (2004). Prenatal dental care: evaluation of professional knowledge of obstetricians and dentists in the cities of Londrire/PR and Bauru/SP, Brazil. J Applied Oral Sci.

[37] **Access to oral health care during the perinatal period: a policy brief.** [http://www.ct.gov/dph/lib/dph/oral_health/pdf/perinatalbrief.pdf]

[38] Enabulele JE, Ibhawoh LO (2014). Knowledge and its sources regarding oral conditions in pregnancy among obstetricians: the need to do more. Trop J Obs Gyn.

